# Influence of marginal bone loss on peri-implantitis: Systematic review of literature

**DOI:** 10.4317/jced.56202

**Published:** 2019-11-01

**Authors:** Alba Carrasco-García, Lizett Castellanos-Cosano, José-Ramon Corcuera-Flores, Antonio Rodríguez-Pérez, Daniel Torres-Lagares, Guillermo Machuca-Portillo

**Affiliations:** 1 PhD student. School of Dentistry, University of Seville; 2 Associate Professor. Oral Surgery, School of Dentistry, University of Seville. University of Fernando Pessoa Canarias; 3 Associate Professor, Oral Surgery, School of Dentistry, University of Seville; 4 Full-time Professor, School of Dentistry, University of Fernando Pessoa Canarias; 5 Full-time Professor, School of Dentistry, University of Seville; 6 MD, DDS, PhD, Professor and Chairman of Special Care Dentistry, School of Dentistry, University of Seville, Spain

## Abstract

**Background:**

The marginal bone of dental implants is subjected to slight load modifications over time, conditioning implant survival. Objective: Perform a systematic review of the literature analyzing the factors that contribute to marginal bone loss (MBL) and the subsequent development of peri-implantitis.

**Material and Methods:**

Bibliographic research in the databases PubMed, Medline and Scopus between 2010 and 2018 was performed. The inclusion criteria were articles published in the last 10 years and that were in English or Spanish, that were carried out on humans, that were cohort studies, that included cases and controls or that used randomized clinical trials. Exclusion criteria removed articles that contained clinical cases, case series or systematic reviews.

**Results:**

A total of 90 articles were analyzed that examined all the factors reported in the literature, such as idiosyncratic factors, toxic habits, systemic drugs and implant characteristics (diameter, length, type surface, implant connection, implant design and type of platform at the moment of the prosthetic load). Discussion: Patient characteristics and associated pathologies must be taken into account when assessing MBL. MBL in all dental implants can be considered independent of the type of prosthetic rehabilitation and the moment of load; this was emphasized. The MBL is smaller in dental implants with rough surfaces, switch platforms and infracrestal localization, as they are of multifactorial origin.

**Conclusions:**

All the reviewed articles maintain a common criterion regarding the concept and measurement of the MBL and highlighting the importance of radiodiagnosis for quantification. Longterm prospective studies with unified criteria are needed to reduce bias by identifying the most relevant factors in MBL.

** Key words:**Marginal bone loss, dental implant, peri-implantitis.

## Introduction

Rehabilitation through dental implants has a long history. Recently, the analysis and relevance of bone remodeling shows that rehabilitation occurs from the moment of dental implant placement and the osseointegration process and from the implant’s subsequent prosthetic rehabilitation and submission to masticatory loads, as well as the conservation and modification of the soft tissues that surround dental implants to achieve adequate function (1).

The study of these variables begins with Albrektsson *et al.* 1986 (2) and continues with Misch *et al.* 2008 (3), who showed how remodeling of the bone surrounding the crestal area of the implant takes place during the first year after implant placement. A loss of up to 2 mm of bone around the neck has been considered to be normal. However, the subsequent remodeling of the surrounding bone must continue be evaluated, since it can ultimately lead to the loss of the dental implant.

To assess a dental implant’s success, different criteria have been described, some of which are still valid today: absence of peri-implant radiolucency, absence of mobility, annual bone loss of less than 0.2 mm after the first year and absence of pain, infection and paresthesias (2). However, new criteria have been incorporated in an attempt to establish scales of implant quality by establishing groups: Group I includes optimal health conditions, Group II includes satisfactory health with stable implants and a history of clinical problems, Group III includes those who have implants with compromised health and Group IV implants are considered failures (3).

Many factors that may influence marginal bone loss (MBL) have been described in the scientific literature: systemic factors of the patient (patient’s baseline pathology, toxic habits), local factors (history/presence of periodontal disease, poor oral hygiene, quality and bone quantity) and characteristics of the implant (surface, diameter, length and morphology) (4-8).

This systematic review aimed to examine previous articles published in the scientific literature that examine which factors contribute to MBL and the development of peri-implantitis.

## Material and Methods

This work has been carried out in accordance with the Preferred Reporting Items for Systematic Reviews and Meta-Analyzes (PRISMA) statement published in 2009 (9). The PI-CO question was asked: What factors influence the initial MBL after the placement of a dental implant and its progression over time? To answer this, a bibliographic search was carried out using PubMed, Medline and Scopus databases and was limited to works published during 2010-2018. The keywords used for the search were “Marginal bone loss,” “dental implant” and “peri-implantitis.”

The inclusion criteria for the research literature were articles published in the last 10 years and that were in English or Spanish, that were carried out on humans and that included the following types of studies: cohort studies, cases and controls or randomized clinical trials.

Exclusion criteria removed articles that contained clinical cases, case series or systematic reviews.

All the information was obtained from the articles selected by one of the authors (ACG). The variables included general information, such as the author, year of publication, type of study, sample size, number of patients evaluated and number of implants placed. Specific variables included the definition used by the authors for MBL, which types of radiography were used for the analysis of MBL, which factors were evaluated in the study and the results obtained.

## Results

**Figure 1:**
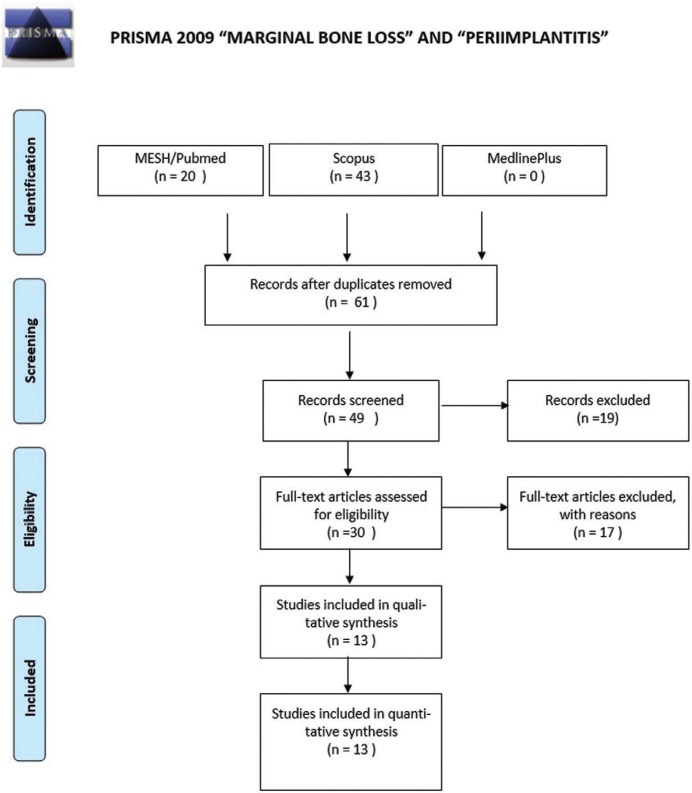
Flowchart with the keywords “marginal bone loss” and “peri-implantitis.”

**Figure 2:**
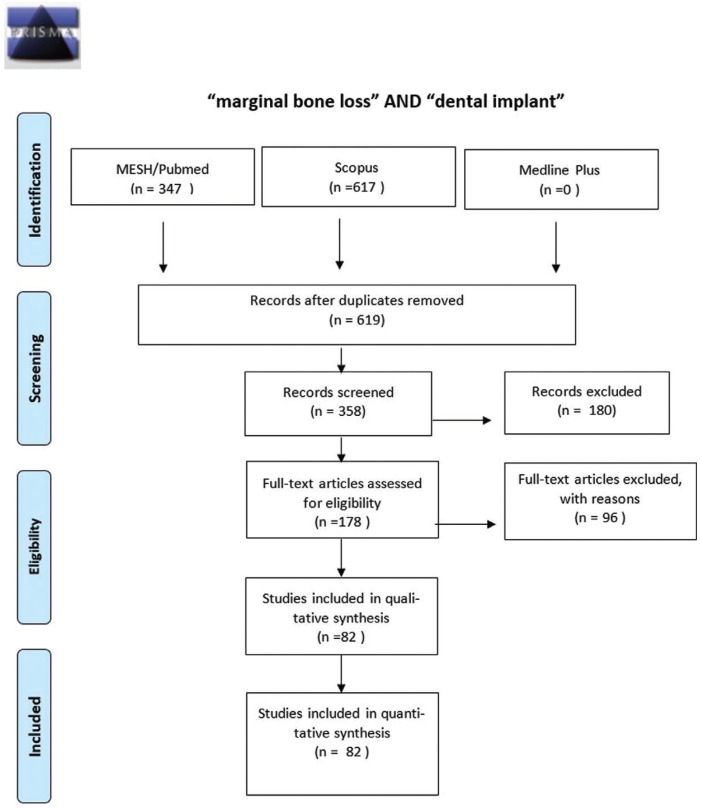
Flowchart with the keywords “marginal bone loss” and “dental implant.”

A systematic research of the PubMed database was carry out with the following research strategies:

1. ((marginal[All Fields] AND (“bone diseases, metabolic”[MeSH Terms] OR (“bone”[All Fields] AND “diseases”[All Fields] AND “metabolic”[All Fields]) OR “metabolic bone dis-eases”[All Fields] OR (“bone”[All Fields] AND “loss”[All Fields]) OR “bone loss”[All Fields])) AND (“peri-implantitis”[MeSH Terms] OR “peri-implantitis”[All Fields] OR “pe-riimplantitis”[All Fields])) AND ((Clinical Trial[ptyp] OR Observational Study[ptyp] OR Con-trolled Clinical Trial[ptyp]) AND “2008/12/01”[PDat]: “2018/11/27”[PDat] AND “hu-mans”[MeSH Terms] AND (English[lang] OR Spanish[lang])). A total of 61 articles were found, of which, after reading the title and summary, 36 were excluded for not complying with the established inclusion criteria; 13 articles were selected (Fig. 1).

2. ((marginal[All Fields] AND (“bone diseases, metabolic”[MeSH Terms] OR (“bone”[All Fields] AND “diseases”[All Fields] AND “metabolic”[All Fields]) OR “metabolic bone dis-eases”[All Fields] OR (“bone”[All Fields] AND “loss”[All Fields]) OR “bone loss”[All Fields])) AND (“dental implants”[MeSH Terms] OR (“dental”[All Fields] AND “implants”[All Fields]) OR “dental implants”[All Fields] OR (“dental”[All Fields] AND “implant”[All Fields]) OR “dental implant”[All Fields])) AND ((Clinical Trial[ptyp] OR Observational Study[ptyp] OR Controlled Clinical Trial[ptyp]) AND “2008/12/01”[PDat]: “2018/11/27”[PDat] AND “humans”[MeSH Terms] AND (English[lang] OR Spanish[lang])). A total of 619 articles were obtained, and after reading the title and abstract, 82 articles were selected (Fig. 2).

After the analysis of the articles obtained from both research attempts, five repeated articles were found, resulting in 90 articles being analyzed.

The articles included in the systematic review of the literature with the research topic of “marginal bone loss and peri-implantitis” and with the research “marginal bone loss and dental implant” can be observed in Table  Table 1,  Table 1 continue,  Table 1 continue-1-18. .

**Table 1:**
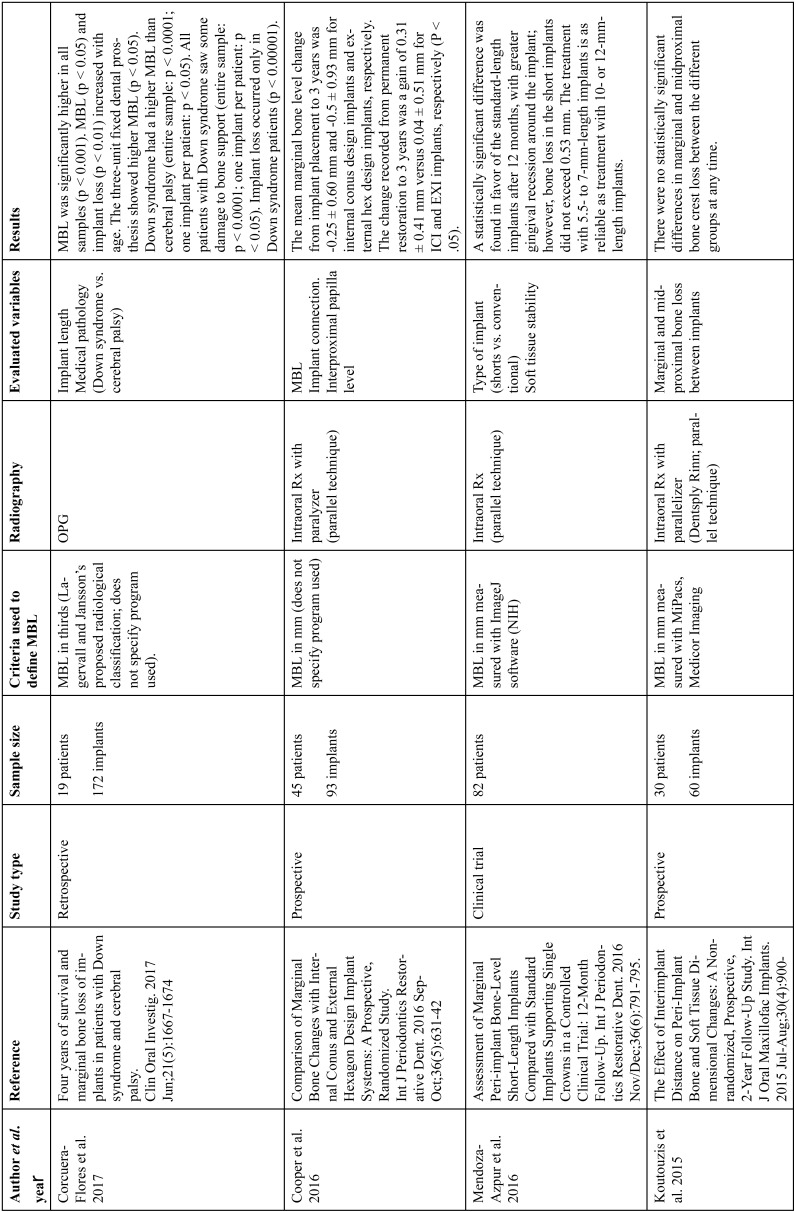
Studies included after the research “marginal bone loss and peri-implantitis” and mar-ginal bone loss and dental implant.

**Table 1 continue:**
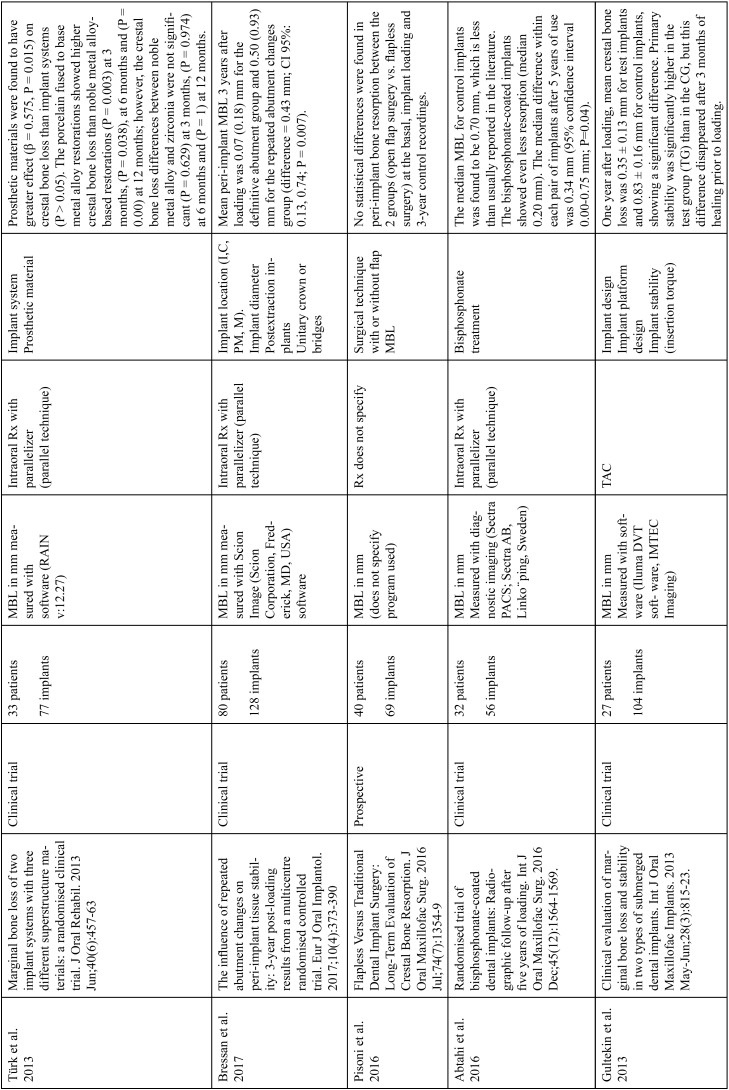
Studies included after the research “marginal bone loss and peri-implantitis” and mar-ginal bone loss and dental implant.

**Table 1 continue-1:**
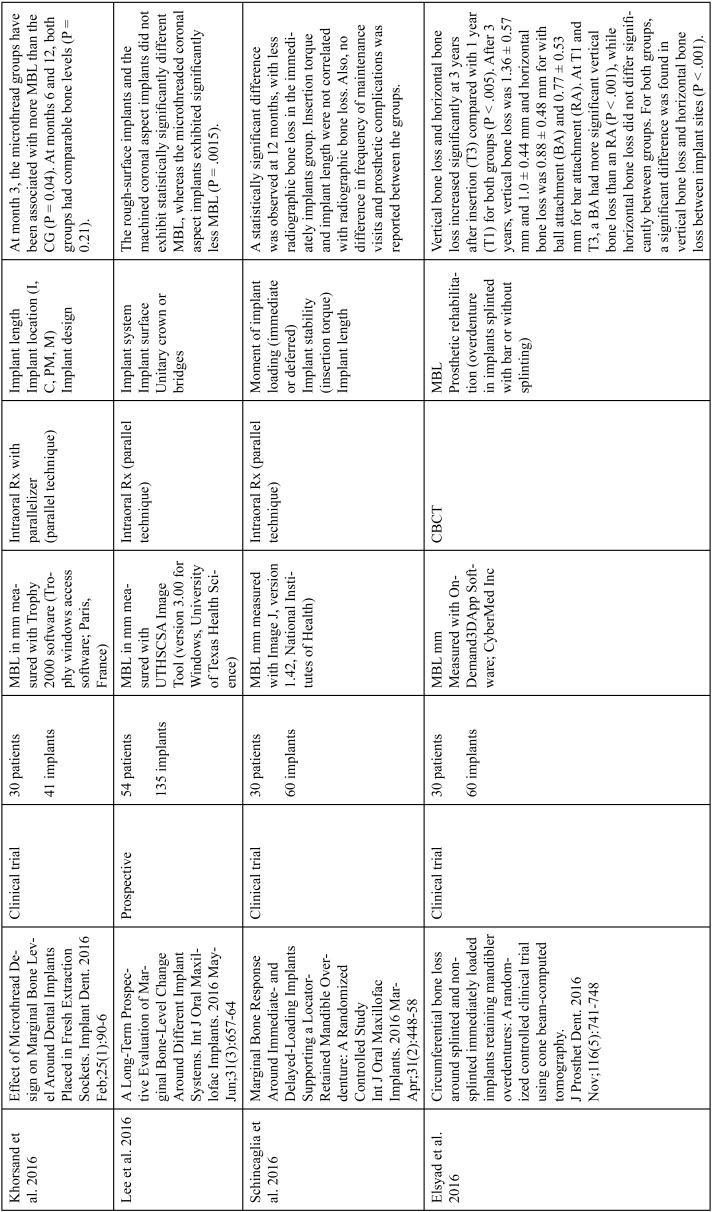
Studies included after the research “marginal bone loss and peri-implantitis” and mar-ginal bone loss and dental implant.

**Table 1 continue-2:**
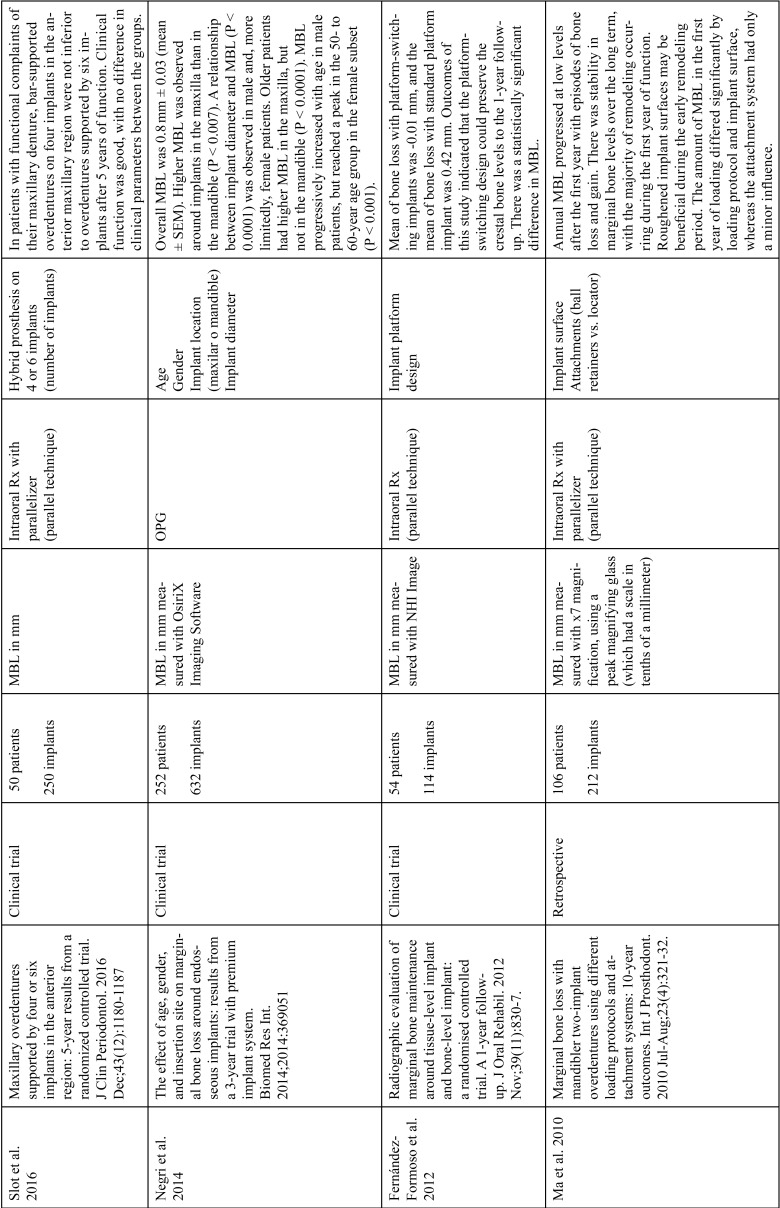
Studies included after the research “marginal bone loss and peri-implantitis” and mar-ginal bone loss and dental implant.

**Table 1 continue-3:**
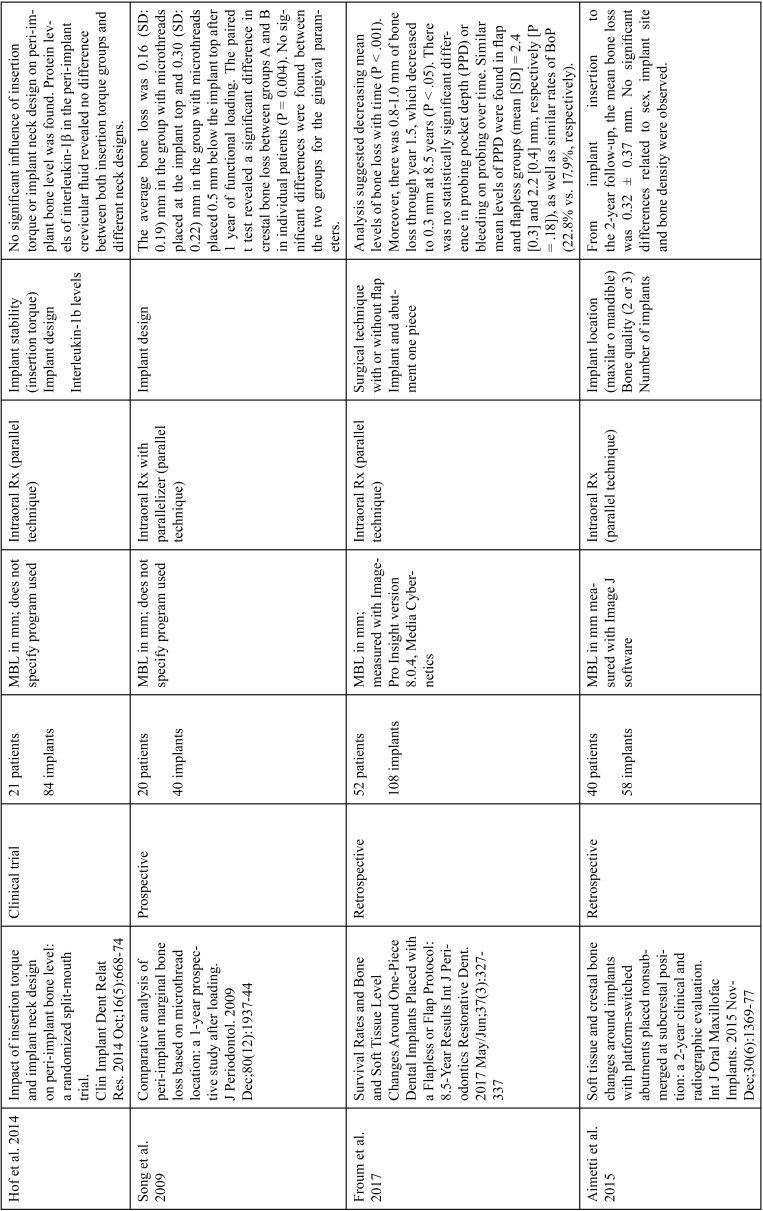
Studies included after the research “marginal bone loss and peri-implantitis” and mar-ginal bone loss and dental implant.

**Table 1 continue-4:**
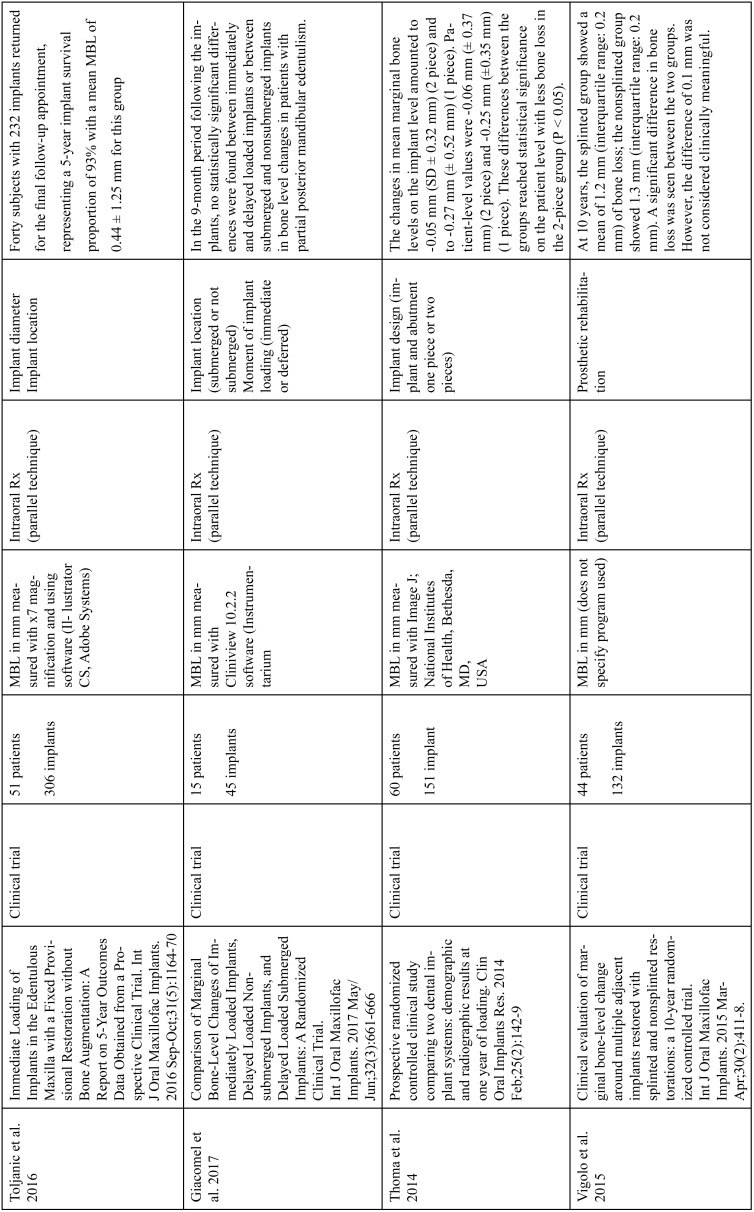
Studies included after the research “marginal bone loss and peri-implantitis” and mar-ginal bone loss and dental implant.

**Table 1 continue-5:**
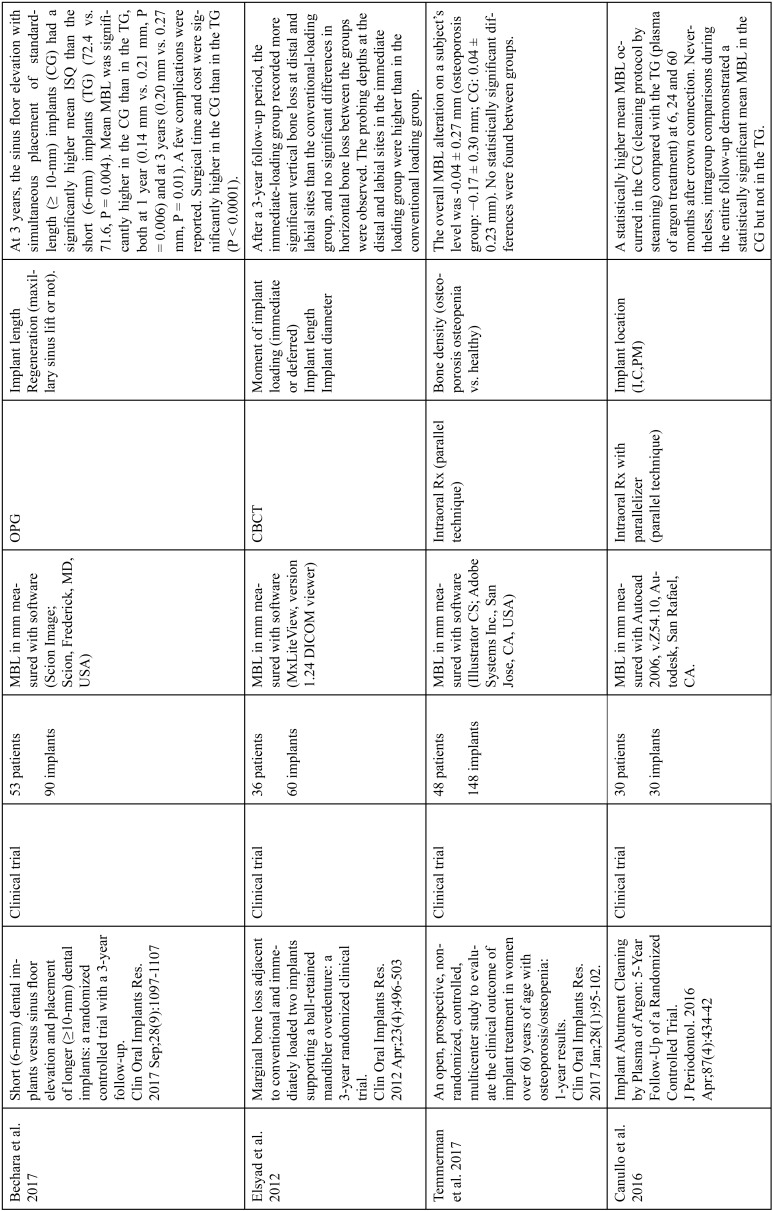
Studies included after the research “marginal bone loss and peri-implantitis” and mar-ginal bone loss and dental implant.

**Table 1 continue-6:**
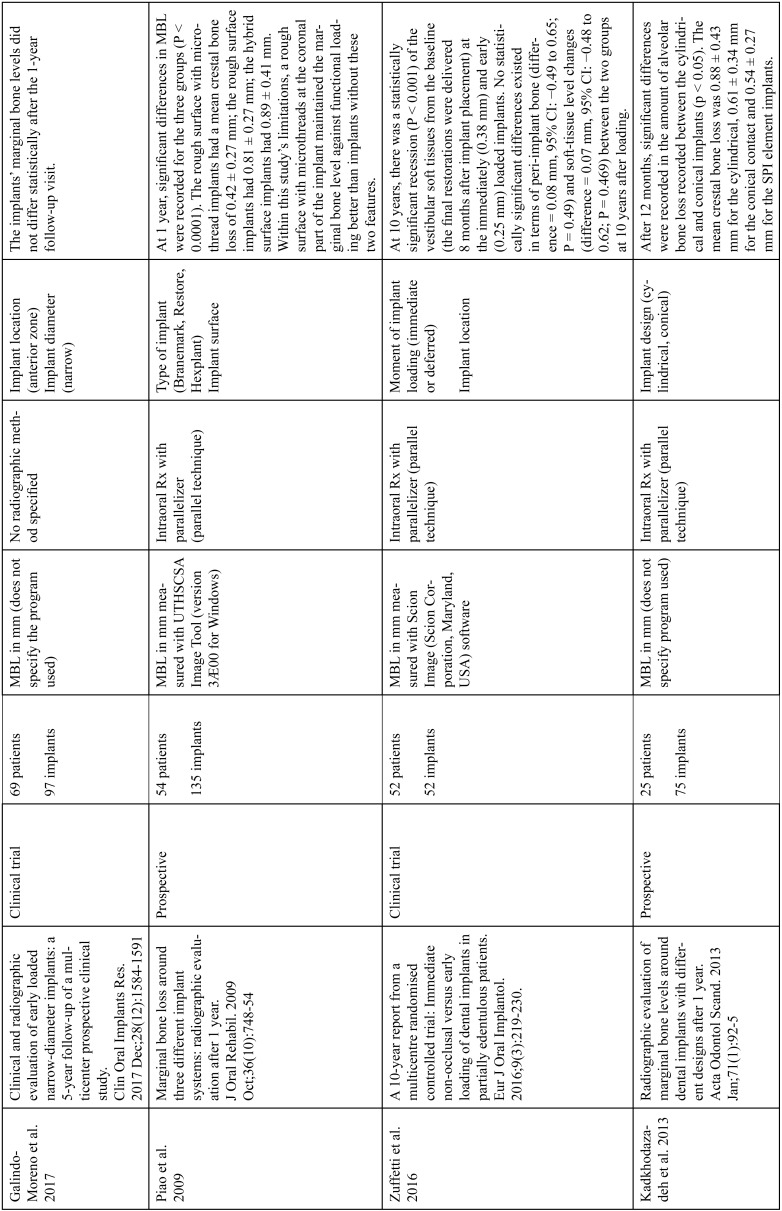
Studies included after the research “marginal bone loss and peri-implantitis” and mar-ginal bone loss and dental implant.

**Table 1 continue-7:**
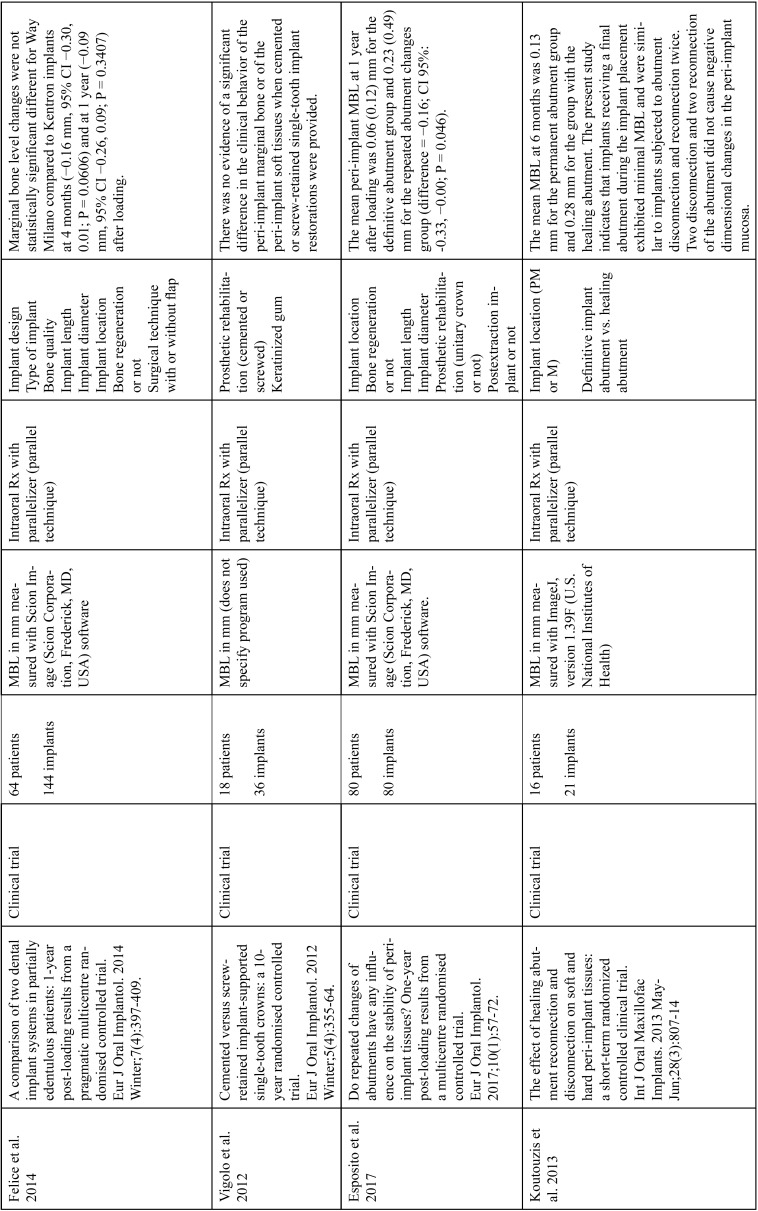
Studies included after the research “marginal bone loss and peri-implantitis” and mar-ginal bone loss and dental implant.

**Table 1 continue-8:**
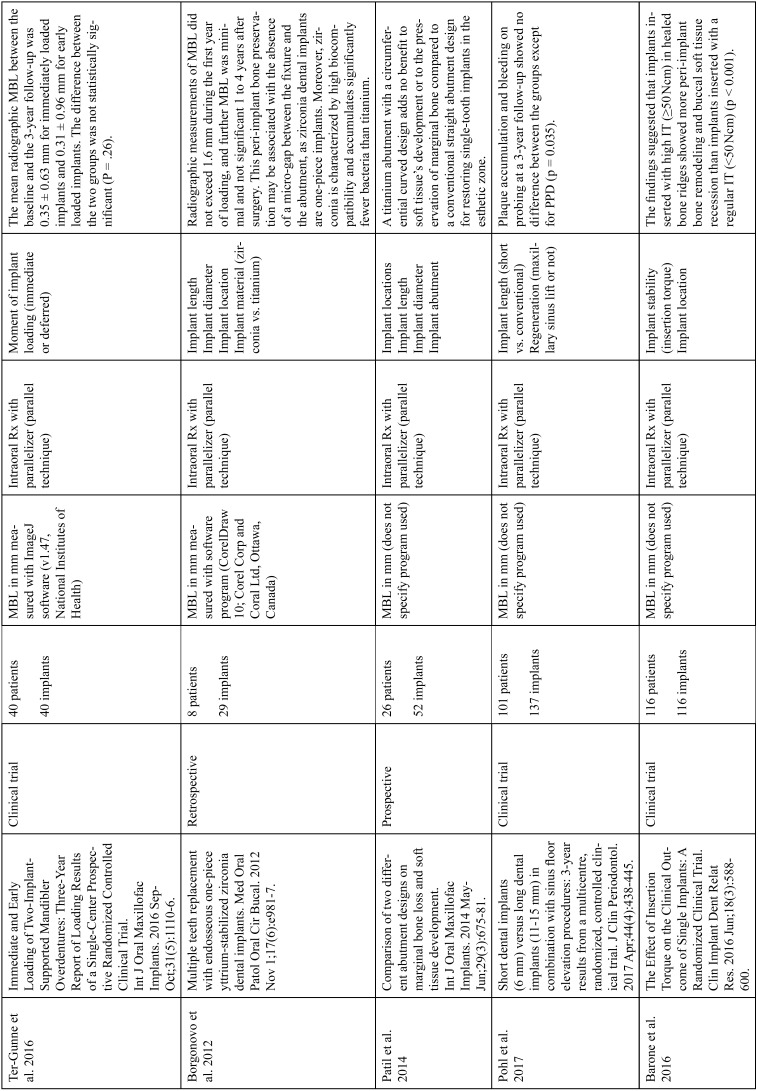
Studies included after the research “marginal bone loss and peri-implantitis” and mar-ginal bone loss and dental implant.

**Table 1 continue-9:**
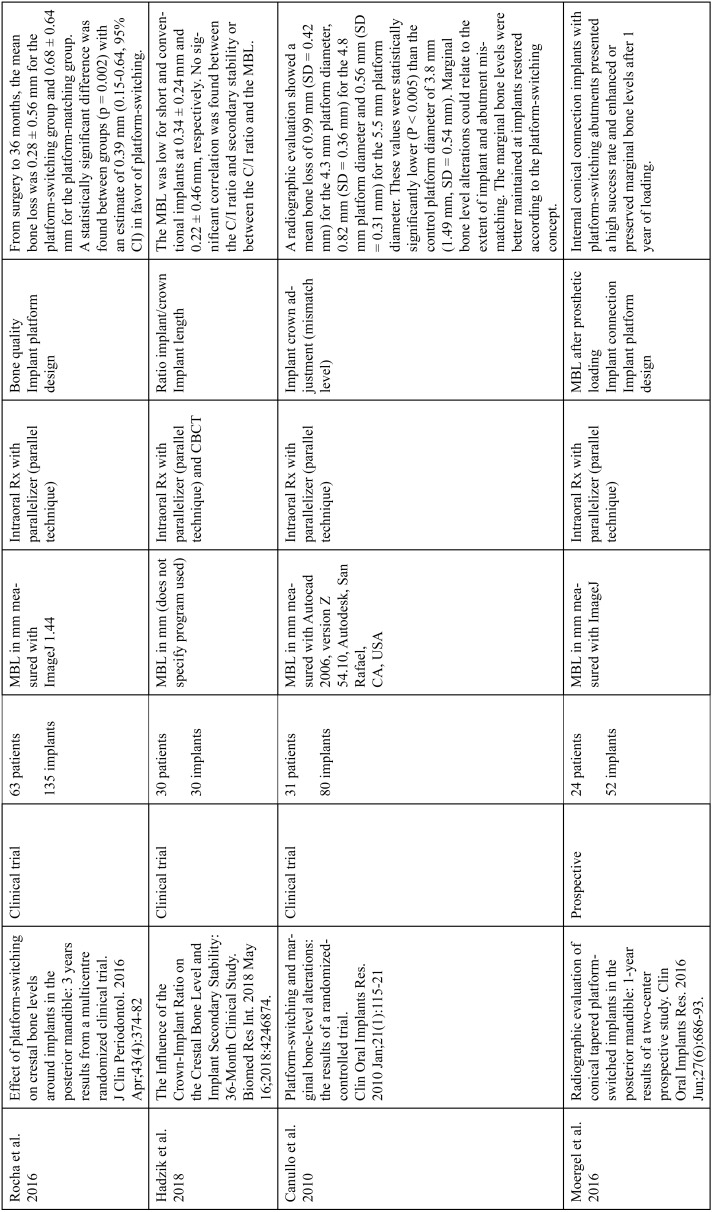
Studies included after the research “marginal bone loss and peri-implantitis” and mar-ginal bone loss and dental implant.

**Table 1 continue-10:**
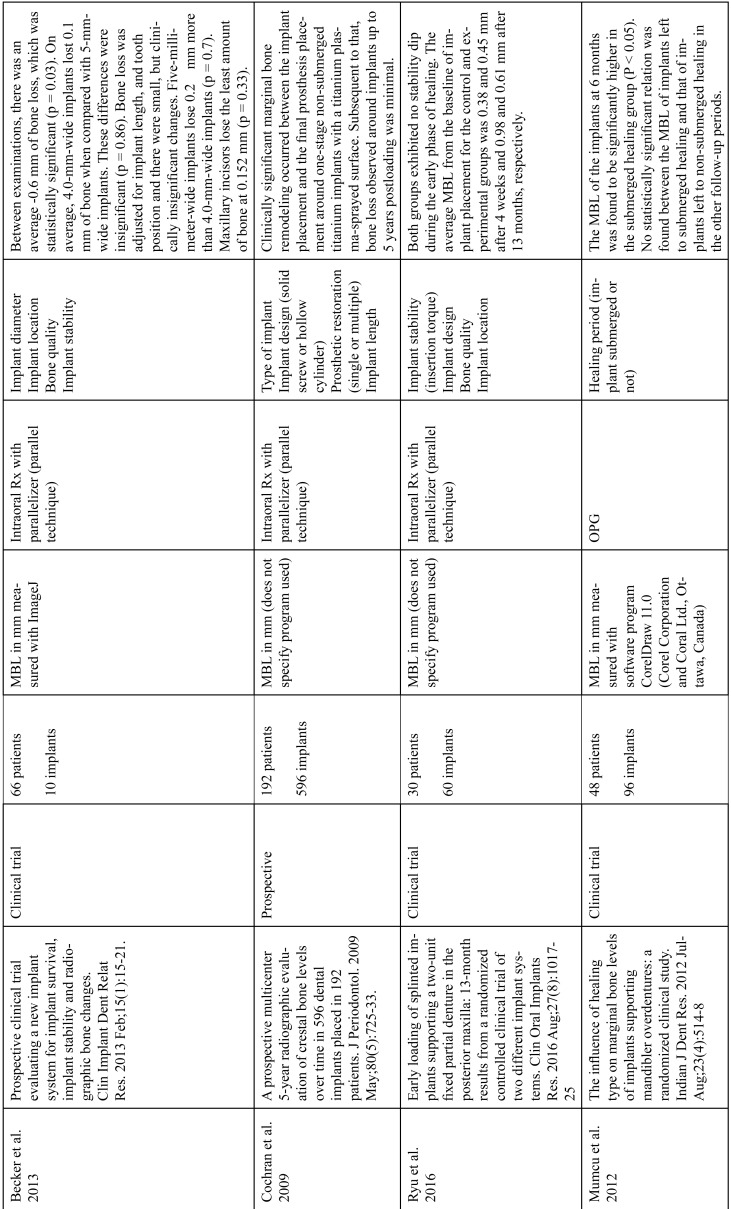
Studies included after the research “marginal bone loss and peri-implantitis” and mar-ginal bone loss and dental implant.

**Table 1 continue-11:**
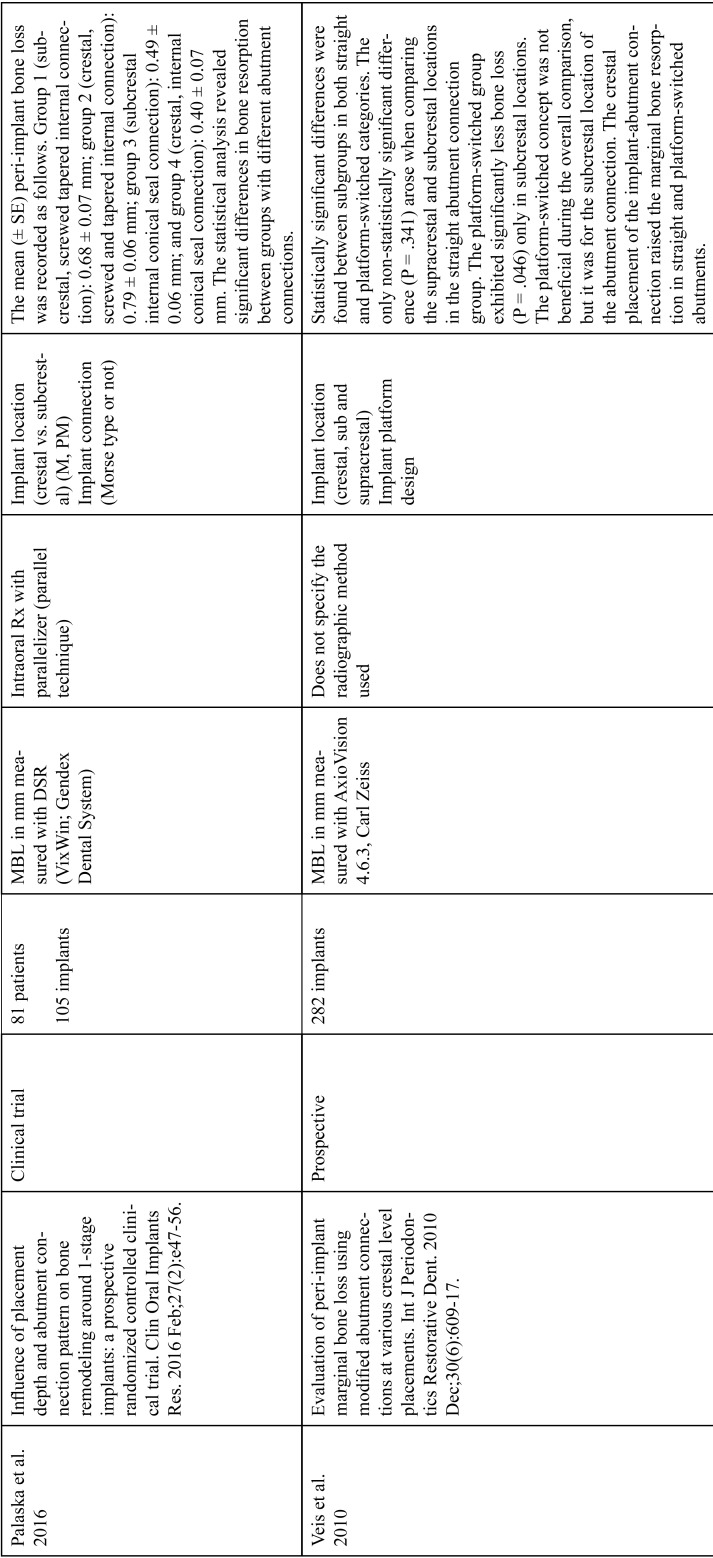
Studies included after the research “marginal bone loss and peri-implantitis” and mar-ginal bone loss and dental implant.

**Table 1 continue-12:**
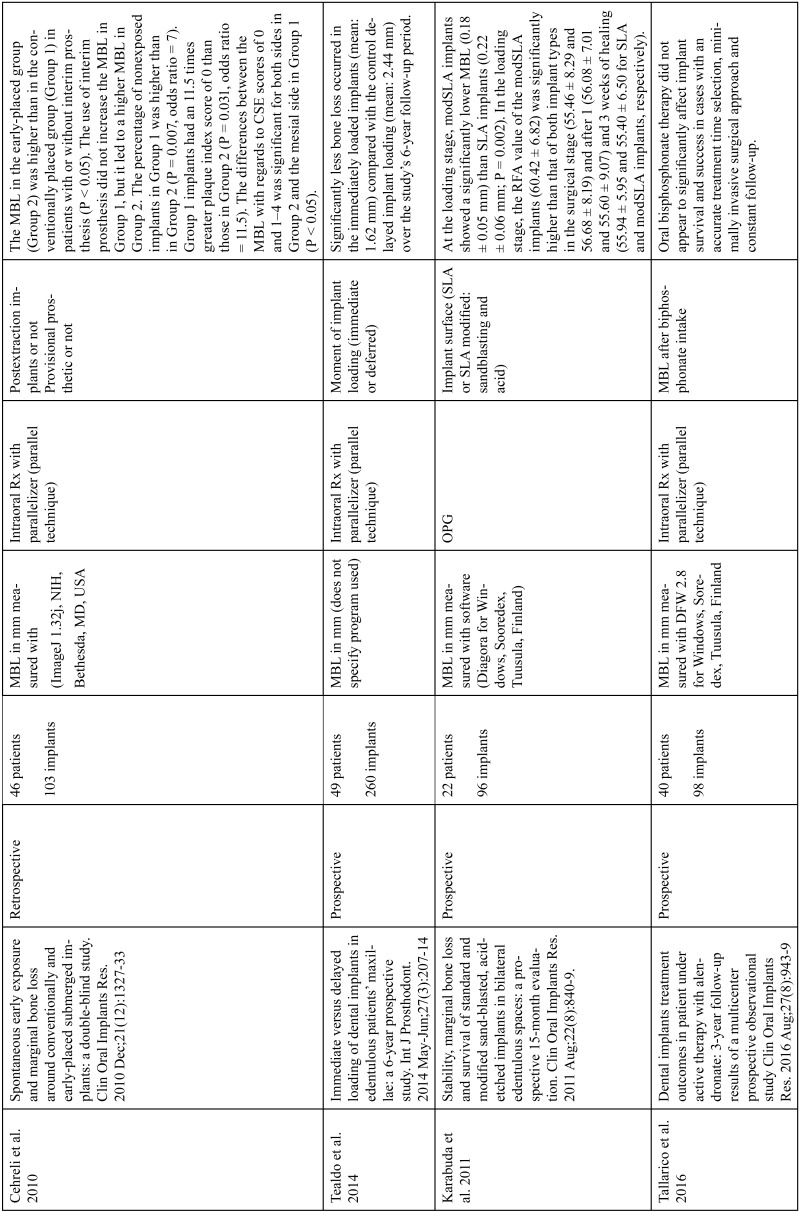
Studies included after the research “marginal bone loss and peri-implantitis” and mar-ginal bone loss and dental implant.

**Table 1 continue-13:**
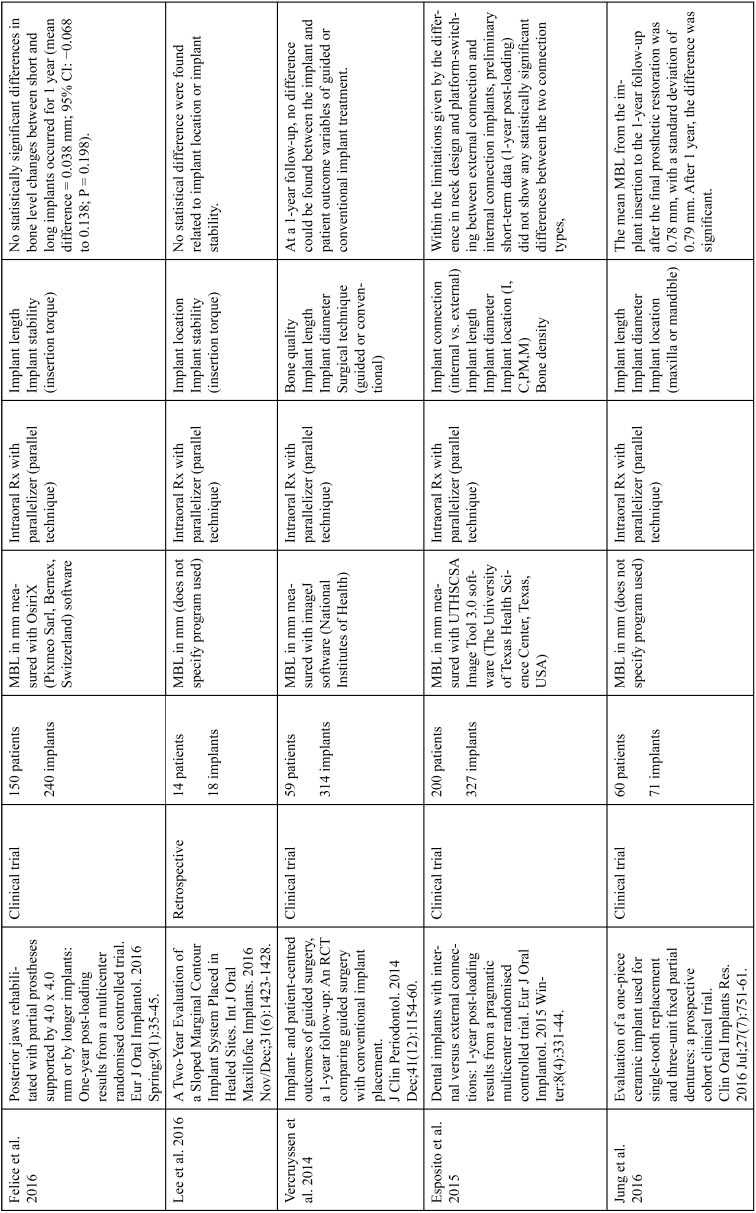
Studies included after the research “marginal bone loss and peri-implantitis” and mar-ginal bone loss and dental implant.

**Table 1 continue-14:**
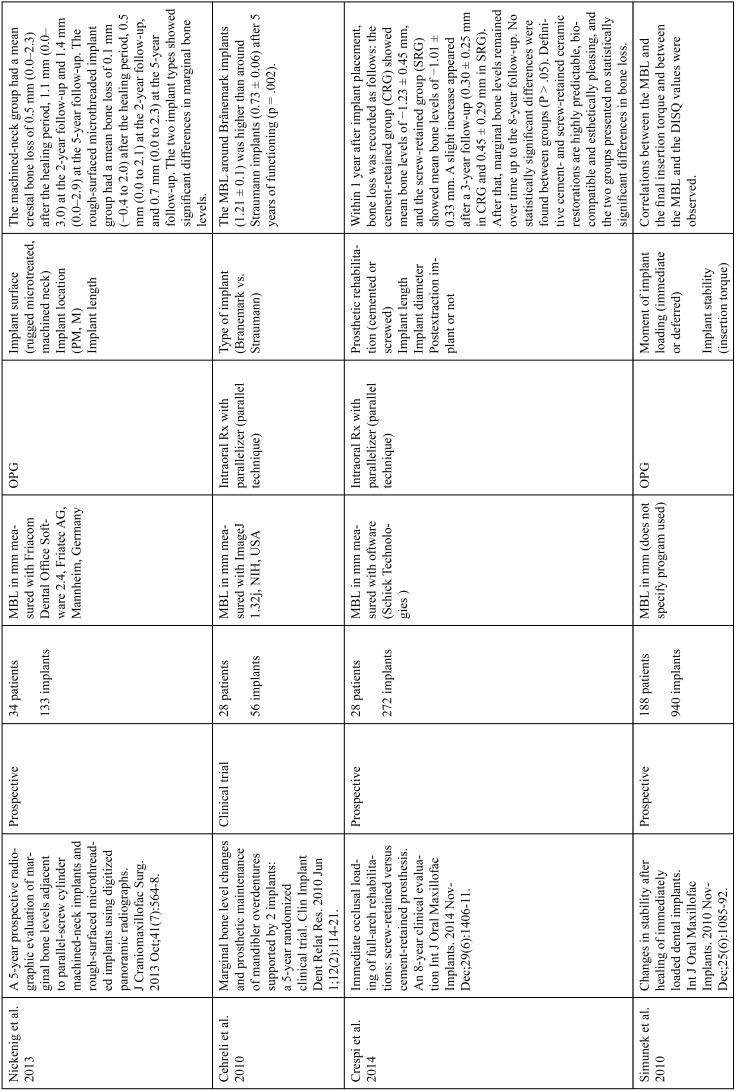
Studies included after the research “marginal bone loss and peri-implantitis” and mar-ginal bone loss and dental implant.

**Table 1 continue-15:**
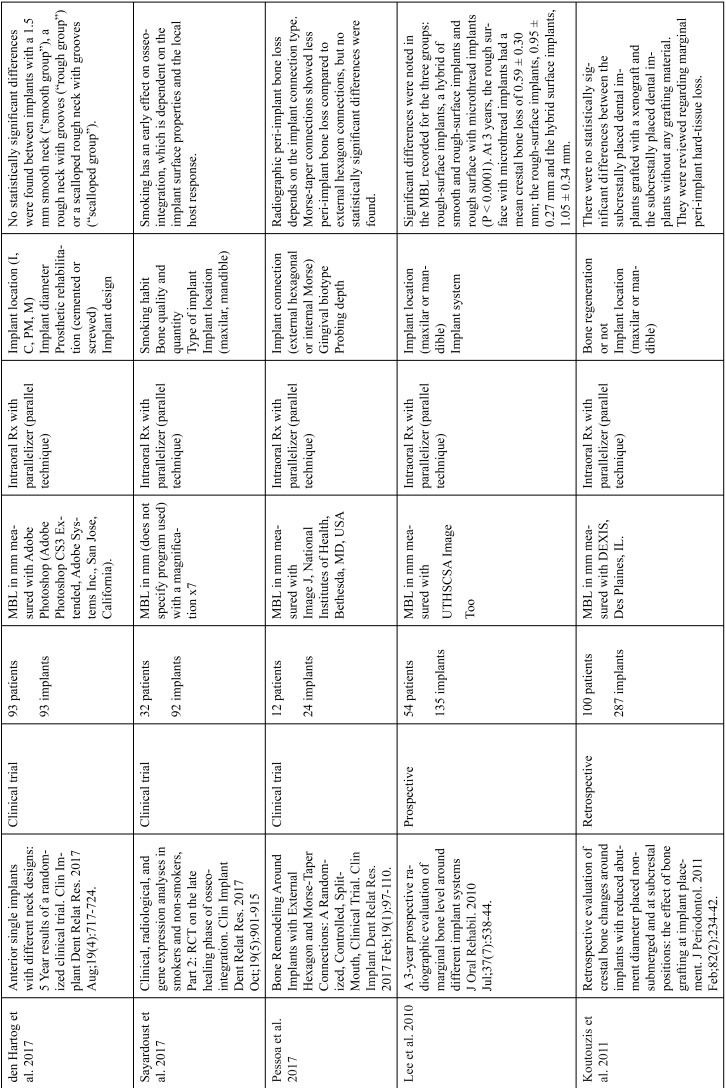
Studies included after the research “marginal bone loss and peri-implantitis” and mar-ginal bone loss and dental implant.

**Table 1 continue-16:**
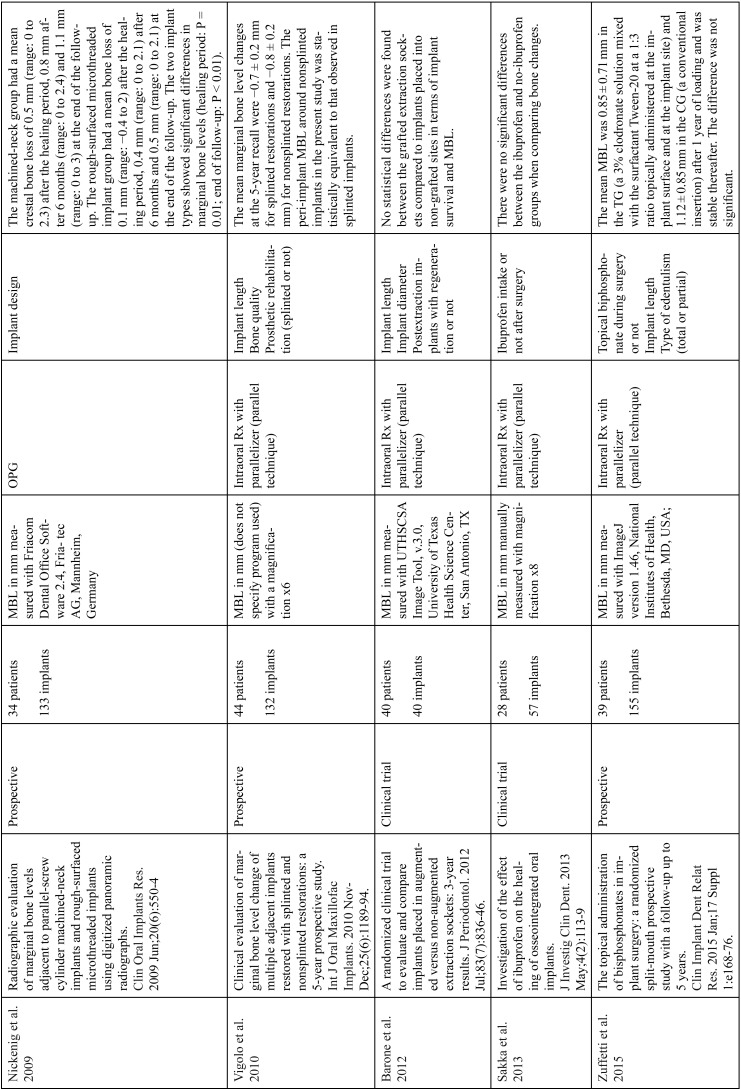
Studies included after the research “marginal bone loss and peri-implantitis” and mar-ginal bone loss and dental implant.

**Table 1 continue-17:**
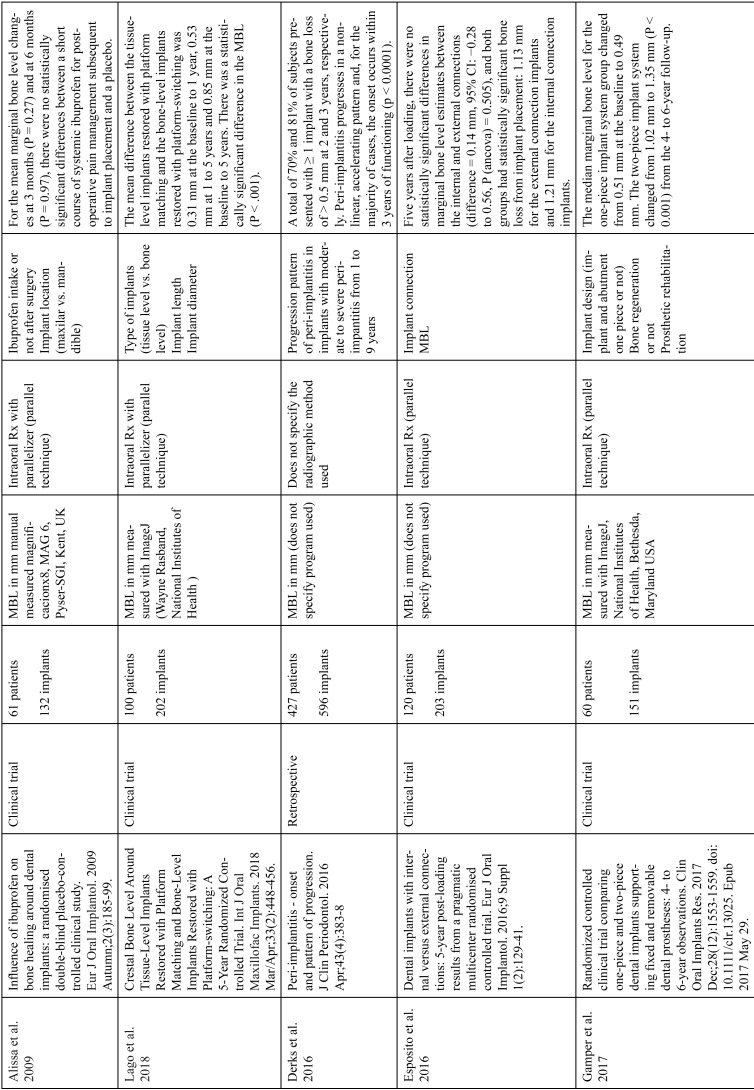
Studies included after the research “marginal bone loss and peri-implantitis” and mar-ginal bone loss and dental implant.

**Table 1 continue-18:**
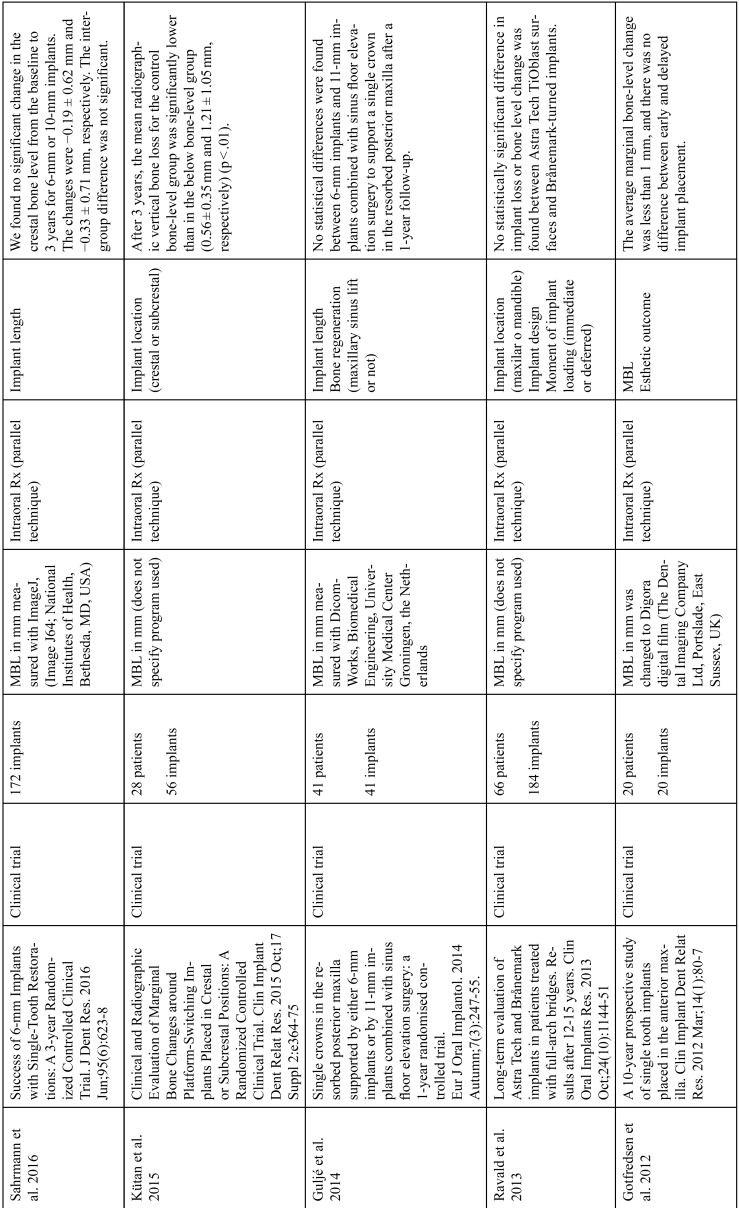
Studies included after the research “marginal bone loss and peri-implantitis” and mar-ginal bone loss and dental implant.

## Discussion

Peri-implantitis was initially described as an infection with pathological changes in the peri-implant tissues (10). Subsequently, different criteria have been defined for its diag-nosis: bleeding, probing depth of higher than 5 mm, exposure of 3 turns or more of the implant’s surface (11) or radiographic observation of MBL, which is defined as bone lost around the crestal area of the dental implant (12).

-MBL Concept 

Several authors defined MBL as the position of the marginal bone based on the position of the crestal bone that is in contact with the implant compared to the more coronal crestal reference point of the implant. Therefore, the MBL measured radiographically mesially and distally would be the vertical distance from the crestal reference point of the implant to the first contact with the bone with the implant at the axial level and is measured parallel to it (13).

The dimensional stability of the tissues is analyzed one year after the prosthetic load of the implant, since during the first year, different authors establish MBL limits of 1.5-2 mm (14), determining a single value of 1.8 mm (15) against the 1.5 mm subsequently established (16). Some authors suggested that after the first year, an MBL greater than 0.2 mm per year can take place (2).

-MBL Diagnostic Tests

For the proper assessment of MBL around dental implants, it is essential to go through complementary diagnostic tests (3). Among the most important radiological tests, we observed the panoramic radiography or orthopantomography and the periapical radiography; these tests will only allow researchers to measure the proximal (mesial and distal) MBL of the implant vertically (3). On the other hand, the conical beam tomography (CBCT) offers us transversal cuts, giving us the possibility to obtain a threedimensional evaluation of the MBL and measure the MBL of the vestibular and lingual area of the implant aside from being able to obtain values in the horizontal sense (17). In addition, with respect to axial tomography, the exposure time, radiation dose and economic cost are reduced (18). However, only 4 of the 90 included studies used cone beam tomography, and 8 of them resorted to panoramic radiography, whereas the vast majority carried out periapical radiographs of the implants by performing a parallel technique with the help of positioners ( Table 1,  Table 1 continue,  Table 1 continue-1-18).

-MBL Quantification 

When quantifying MBL, some authors used different software (19) and even used magnifying glasses to be more exhaustive with the measurement (20). All the studies included in this review expressed this MBL quantitatively in millimeters, except for the study performed by Corcuera *et al.* 2016 (21), in which Lagervall and Jansson’s classification was used (22). This classification gives grade 0 if there is no MBL, grade 1 when the loss is equal to or less than 1/3 of the length of the implant, grade 2 if the loss is greater than 1/3 but less than 2/3 of the length of the implant and grade 3 if it suffers 2/3. Corcuera *et al.* 2016 made a modification to this classification, incorporating a grade 4 that includes implants that were unsuccessful or non-surviving (21).

-MBL Influential Epidemiological Factors 

The main idiosyncratic patient factors recorded in the studies reviewed were age, gender, toxic habits and systemic pathology (osteoporosis, osteopenia, Interleukin-1b levels), in addition to the medication administered (bisphosphonates) (23-27).

Few studies focused on assessing MBL and highlighted the presence of systemic pathology, including a patient’s medication and/or their toxic habits. Although greater MBL has been observed in smoking patients, independently of the type of implant rehabilitation, MBL sometimes duplicates these results independently of the type of implant rehabilitation applied (28). Sayardoust *et al.* 2017 (25) indicated that MBL was higher in the mechanized implants of smoking patients at 90 days, with a higher expression of the proinflammatory cytokine IL-6 and a lower expression of the osteogenic and osteocalcin gene. Predictors of MBL are reflected in smoking, bleeding as a result of probing at 90 days, an expression of factor 1 alpha and an expression of proinflammatory cytokine at 90 days (25).

Corcuera *et al.* 2017 (21) concluded that patients with Down syndrome have significantly higher MBL (*p* < 0.001) if one implant per patient is selected (*p* < 0.05). They also observed a greater loss of implants, especially in those with greater MBL (*p* < 0.01). In the case group, an increase in MBL (*p* < 0.05) and greater implant loss (*p* < 0.01) was also observed with age.

Temmerman *et al.* 2017 (27) focused their study on postmenopausal women with osteoporosis/osteopenia, showing an MBL around implants of 0.01 ± 0.51 mm (Control Group [CG]: 0.05 ± 0.52 mm); the mean MBL from a subject was of 0.04 ± 0.27 mm (Osteoporosis/Osteopenia Group: ˗0.17 ± 0.30 mm, CG: 0.04 ± 0.23 mm). They concluded that rehabilitation using implants in patients with osteoporosis can be carried out with the same success rate as in healthy patients.

The stability analysis off dental implants can also be conditioned due to certain drugs that treat the patient’s underlying pathology and that alter bone remodeling immunity (28). Bisphosphonates are one such drug family that has been studied due to their ability to inhibit normal bone resorption, which entails a reduction in remodeling, for a higher bone density, better mineralization and a lower risk of bone fracture (29). Tallarico *et al.* 2016 (26) found that patients who were previously under bisphosphonate treatment had a 98.98% overall implant survival success rate and 100% prosthesis success rate. The mean MBL was 1.35 ± 0.21 mm (95% CI 1.24-1.38) at 3 years. Zuffetti *et al.* 2015 (30) obtained 100% survival rates in the implants for the experimental group; the implants for this group were topically rinsed with bisphosphonate before surgery implantation. The CG, on the other hand, had a 91.3% success rate; in this group, the implants were placed without applying the topical bisphosphonate rinse, and a significant difference was observed at 5 years (*p* < 0.01). The average MBL observed was 0.85 ± 0.71 mm in the experimental group and 1.12 ± 0.85 mm in the CG after one year of prosthetic loading; it then remained stable with no statistically significant differences. Thus, some authors suggested that implants coated with bisphosphonates allow the prolonged conservation of marginal bone. These authors observed that the implants coated with bisphosphonates show even less marginal bone resorption than the CG not coated with bisphosphonates, and a mean difference after 5 years of loading 0.34 mm (95% confidence interval 0.00-0.75 mm, *p* = 0.04) was observed (23).

The medication prescribed after surgery must also be taken into account due to its possible effect on MBL, since several studies observed a greater MBL in patients who were administered ibuprofen after surgery, although their results were not statistically significant (20).

-MBL Factors Related to Implant Design 

Dental implant surface is one of the factors more frequently analyzed, a greater crestal bone loss was observed in those implants with a mechanized surface (p = 0.01, end of follow-up: *p* < 0.01) (31). Among implants with a rough surface, those with coronal microtreatments have the lowest MBL compared with rough surface and hybrid implants (*p* < 0.05) (32,33). Other authors agreed with these results, although they did not observe any statistically significant differences between the rough surface implants and the implants with mechanized coronal parts (34). However, the type of surface treatment used by different brands of implants with rough surfaces has been found to have lower MBL (*p* = 0.002) (35). However, they suggested that implants with a microtreatment in the crestal area of the implant may not have a positive effect on implants located in the anterior maxilla and with relatively recent extractions (36).

In addition to considering the surface of an implant, implant design has been studied, comparing cylindrical implants and conical implants; these studies have concluded that the implants with less highly polished surfaces and cylindrical shapes suffered less MBL per year in a statistically significant way (*p* < 0.05) (37). The extension of the spirals along the implant is another feature that has been studied and that shows a statistically significant difference in crestal bone loss, specifically between implants designed with spirals that run until the coronal part of the implant, which suffered a loss of 0.16 mm (SD 0.19 mm) after one year of functional loading compared to 0.30 mm (SD 0.22 mm) in the implants without spirals reaching the coronal part (38).

Implant platform and connection type to the prosthetic abutment have also been shown to influence MBL. The implant abutment connection being located in the crestal area in straight implants and implants with platform-switching reflects greater marginal bone resorption, and the implants with platform change in infracrestal locations showed a significantly lower bone loss (8). This suggests that platform-switching can facilitate bone conservation at the crestal level (4). The implants with platform-switching and conical connection presented MBL that was significantly lower than implants with a standard platform (5). Some authors highlighted a slightly lower MBL in crestal implants compared with infracrestal implants (*p* < 0.01) (6). However, other authors did not obtain differences in the MBL of infracrestal implants with platform-switching in the maxilla and mandible (7). When analyzing the implant’s location with an internal conical connection screwed into an infracrestal and a crestal position and with respect to implants with a conical connection and a Morse-type internal seal in similar positions, no significant differences were observed, although the group with the crestal location and Morse-type connection presented the lowest MBL (8).

The type of connection between dental implant and prosthetic rehabilitation has also been studied; some studies show that after one year of follow-up, there is significant MBL in implants with internal connection and in implants with external connection without a statistically significant difference existing between the type of connections (39). However, the results obtained in other studies highlight a greater MBL in external connection implants (40).

## Conclusions

The analyzed articles maintain a common criterion regarding the concept and measurement of the MBL, emphasizing the importance of the radiodiagnosis for its quantification. Few articles focus on the relationship between systemic pathology and MBL. In relation to the characteristics of the implants, the conclusions are more unequal and less homogeneous, highlighting an MBL in all the implants, regardless of the type of prosthetic rehabilitation and moment of loading. MBL is lower when an implant’s surface is rough and in implants with platform-switching and infracrestal position. However, greater loss is described in those with external connections. No statistically significant alterations were observed in surgical techniques with a flapneither in one-piece implants nor in the type of crown fixation (screwed versus cemented). Therefore, several factors of different natures can influence MBL, requiring more studies be done to increase our knowledge about how to prevent MBL.
